# Economic evaluation of margetuximab vs. trastuzumab for pretreated ERBB2-positive advanced breast cancer in the US and China

**DOI:** 10.3389/fpubh.2022.942767

**Published:** 2022-09-09

**Authors:** Zhiyuan Tang, Xin Xu, Jie Gao, Ling Chen, Qiuyan Zhu, Jinli Wang, Xiaoyu Yan, Bohua Chen, Yumei Zhu

**Affiliations:** ^1^Department of Pharmacy, Affiliated Hospital of Nantong University, Nantong, China; ^2^School of International Pharmaceutical Business, China Pharmaceutical University, Nanjing, China

**Keywords:** margetuximab, trastuzumab, cost-effectiveness, breast cancer, China, the US

## Abstract

**Objective:**

To assess the economic evaluation of margetuximab plus chemotherapy over trastuzumab plus chemotherapy for women with pretreated ERBB2-positive advanced breast cancer in the United States (US) and China.

**Methods:**

Based on the SOPHIA trial, a three-state Markov model was developed to compare the cost and efficacy of margetuximab to trastuzumab for previously treated women with ERBB2-positive advanced breast cancer. The model inputs were derived from existing literature and the US life table. Primary outcomes included lifetime costs in US dollars, quality-adjusted life-years (QALYs), and incremental cost-effectiveness ratio (ICER). Deterministic and probabilistic sensitivity analyses were conducted to evaluate the impact of uncertainty.

**Results:**

The base case analyses demonstrated that margetuximab plus chemotherapy had an increasing cost of $68,132 and $20,540 over trastuzumab plus chemotherapy in the US and China, respectively, with a gain of 0.11 and 0.09 QALYs both favored margetuximab. The ICERs for two treatment strategies were $260,176 in the US and $630,777 in China, resulting in a poor cost-effectiveness at their respective threshold of willingness to play. One-way sensitivity analyses showed that the results to be most sensitive to the price of margetuximab and that of trastuzumab. And an 11 and 82% price reduction of margetuximab would make this regimen cost-effective in the US and China, respectively.

**Conclusion:**

In the US and China, margetuximab plus chemotherapy is not likely to be cost-effective for women with pretreated ERBB2-positive advanced breast cancer, whereas price reduction effectively improves insufficient cost-effectiveness.

## Introduction

Breast cancer has replaced lung cancer as the most prevalent cancer globally, with 2.26 million new cases worldwide in 2020 (1). Among women, invasive adenocarcinoma of the breast is the most common non-dermatological cancer, with the second and fourth leading cause of death in the United States (US) and China (2, 3). About 6% of breast cancer patients are diagnosed with advanced breast cancer in the US (4), while the rate is more than 20% among Chinese patients (5). Around 20 to 30% of women with breast cancer diagnoses have overexpressed Human epidermal growth factor receptor 2 (ERBB2, formerly HER2), which is associated with more aggressiveness and worse prognosis (6).

The economic burden of breast cancer is increasing rapidly with the changing treatment landscape. The 1-year treatment cost after breast cancer diagnosis increased by 2-fold within 10 years, with approximately an estimate of $20 billion by 2020 (7, 8). A tripling proportion of chemotherapy-received women (9), namely incremental use of new oncolytic drugs, contributes to increased cancer-related costs and pressure on health care budgets. Unfortunately, the higher population of patients with ERBB2-positive late-stage breast cancer will further incur higher cancer-related drug costs (10, 11). For these patients, the standard first-line treatment included trastuzumab plus taxane in the earlier years, and since 2013, the addition of pertuzumab to trastuzumab with taxane became routinely available (12, 13). Despite the marked clinical efficacy of the combination of trastuzumab, pertuzumab, and chemotherapy in patients with first-line advanced breast cancer, the vast majority of patients ultimately progress. Mounting evidence demonstrate that previous-treated patients with progression can still benefit from additional ERRB2-targeted agents, while the optimal treatment paradigm in later lines remains unsettled (14, 15).

Margetuximab, a chimeric, Fc-engineered, immune-activating anti-ERBB2 immunoglobulin G1 monoclonal antibody, shares epitope specificity and Fc-independent anti-proliferative effects with trastuzumab. Based on the SOPHIA phase three randomized open-label trial, margetuximab plus chemotherapy had acceptable safety and significant clinical benefits compared with trastuzumab plus chemotherapy in ERBB2-positive advance breast cancer after two or more prior anti-ERBB2 therapies (16). It significantly prolonged median progression-free survival (PFS) by 1.3 months (5.7 verse 4.4 months, hazard ratio (HR), 0.71; 95% confidence interval (CI), 0.58 to 0.86) and the median overall survival (OS) by 1.8 months (21.6 verse 19.8 months, HR, 0.89, IC, 0.69 to 1.13) for patients receiving margetuximab in comparison to trastuzumab. Owing to the improved efficacy, in 2020, the US Food and Drug Administration (FDA) approved margetuximab in combination with chemotherapy as the treatment of adult patients with metastatic ERBB2-positive breast cancer who have received two or more prior anti-ERBB2 regimens.

Since this treatment regimen exhibited proven effectiveness, there is an impetus for evaluating its economic value. The objective of this model-based analysis was to estimate the potential cost-effectiveness of margetuximab compared to trastuzumab, each combined with chemotherapy, for patients with pretreated ERBB2-positive advanced breast cancer in developed and developing countries, like the US and China.

## Methods

### Study design and setting

To estimate the effectiveness and cost outcomes of patients with pretreated ERBB2-positive advanced breast cancer, this study conducted a Markov model with a 3-week cycle length to compare margetuximab vs. trastuzumab, each with chemotherapy, in the context of the US and Chinese health care system. The base-case intention-to-treat (ITT) population was 56 years and over women who had progressive disease after two or more lines of prior ERBB2-targeted therapy (including pertuzumab), and one to three lines of non-hormonal metastatic breast cancer therapy (16). The model was evaluated based on a time horizon of the rest of a patient's life, alongside a discount rate of 3% per annum in costs and outcomes. Treatment effectiveness was assessed as life-years (LYs) and quality-adjusted life-years (QALYs). Primary economic endpoint was the projected incremental cost-effectiveness ratio (ICER). The willingness to pay (WTP) threshold of $150,000 per QALY in the US and $37,653 per QALY in China (triple GDP per capita) was used to determine cost-effectiveness.

### Simulation model

**Figure 1:**
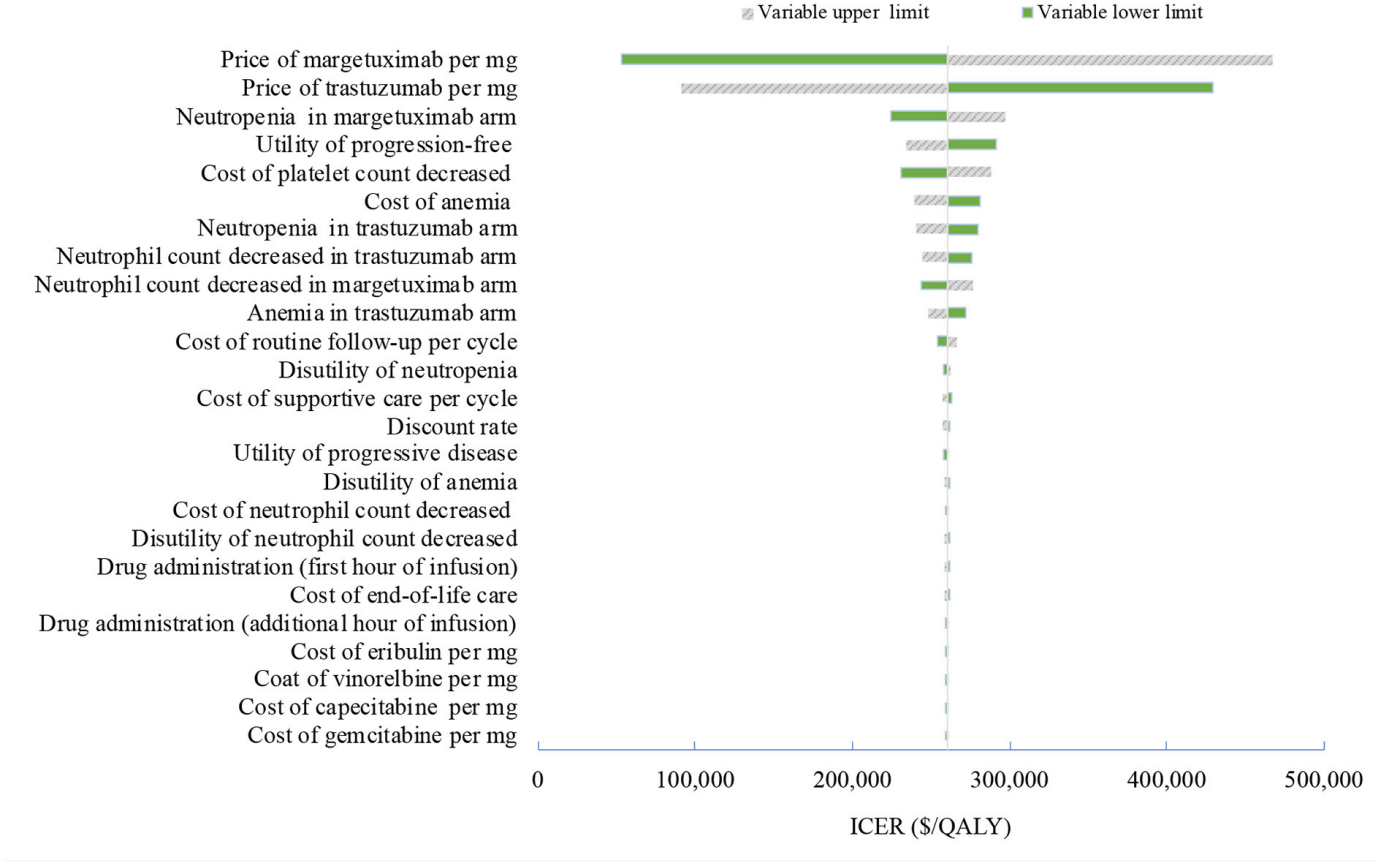
Tornado diagram of one-way sensitivity analyses of margetuximab vs. trastuzumab in the treatment of pretreated ERBB2-positive advanced breast cancer from the US perspective. QALYs, quality-adjusted life-years.

The Markov model included three mutually exclusive health states: PFS, progressed disease (PD), and death (Figure 1). All pretreated patients began in the PFS state and would either remain in their assigned health state or transition to a new health state based on time-dependency transition probabilities during each 3-week cycle. The half-cycle correction was applied to all estimated costs and utilities to avoid reducing the actual cost and effectiveness of loading doses of margetuximab and trastuzumab.

### Clinical data

Data from the SOPHIA trial was used to model the PFS and OS curves. Kaplan-Meier estimates beyond the observation period were extrapolated based on the standard statistical analyses developed by Guyot et al. (17). The study used the GetData Graph Digitizer software (2.21 version) to gather the data points from the PFS and OS curves, fitting parametric survival functions. Multiple parametric distributions included exponential, Weibull, lognormal, gamma, log-logistic, and Gompertz. Goodness-of-fit was assessed according to Akaike Information Criterion and the Bayesian Information Criterion, combined with the visual inspection. The log-logistic distribution was adopted for PFS curves and the Weibull distribution for OS curves (Supplementary Tables S1, S2). The model estimated the mortality rate through the US and Chinese life tables. Health state utilities were sourced from the literature. Table 1 includes a summary of the utility estimates used in the model.

**Table 1:** Model inputs.

**Parameter**	**Base case**	**range**		**Distribution**	**Source**
		**Low**	**High**		
Cost in the US ($)					
Margetuximab per mg	8.75	7.00	10.50	Gamma	2022 ASP
Trastuzumab per mg	8.64	6.91	10.37	Gamma	2022 ASP
Capecitabine per mg	0.00	0.00	0.00	Gamma	2022 ASP
Eribulin per mg	1,274.36	1,019.49	1,529.23	Gamma	2022 ASP
Gemcitabine per mg	0.02	0.02	0.02	Gamma	2022 ASP
Vinorelbine per mg	0.86	0.69	1.03	Gamma	2022 ASP
Supportive care	5,600.00	4,480.00	6,720.00	Gamma	(18)
Routine follow-up	1,890.00	1,512.00	2,268.00	Gamma	(18)
End-of-life care	21,585.00	17,268.00	25,902.00	Gamma	(18)
Drug administration					
First h of infusion	136.61	109.29	163.93	Gamma	(18)
Additional h of infusion	28.71	22.97	34.45	Gamma	(18)
Management of adverse events					
Neutrophil count decreased	10,603.70	8,482.96	12,724.44	Gamma	(19)
Anemia	146,36.53	11,709.22	17,563.84	Gamma	(19)
Neutropenia	10,603.70	8,482.96	12,724.44	Gamma	(19)
Cost in China ($)					
Margetuximab per mg	8.75	7.00	10.50	Gamma	Local price
Trastuzumab per mg	1.94	1.55	2.33	Gamma	Local price
Capecitabine per mg	0.02	0.02	0.03	Gamma	Local price
Eribulin per mg	617.15	493.72	740.58	Gamma	Local price
Gemcitabine per mg	0.09	0.07	0.10	Gamma	Local price
Vinorelbine per mg	2.38	1.90	2.85	Gamma	Local price
Supportive care	1,616.78	1,293.42	1,940.14	Gamma	(20)
Routine follow-up	162.00	129.60	194.40	Gamma	(20)
End-of-life care	1,275.03	1,020.02	1,530.04	Gamma	
Drug administration	22.00	17.60	26.40	Gamma	(21)
Management of adverse events					
Neutrophil count decreased	3,184.01	2,547.21	3,820.81	Gamma	(22)
Anemia	607.52	486.02	729.03	Gamma	(20)
Neutropenia	3,184.01	2,547.21	3,820.81	Gamma	(22)
Risks for main AEs in margetuximab arm (grade ≥3)					
Neutrophil count decreased	0.09	0.08	0.10	Beta	(16)
Neutropenia	0.20	0.18	0.22	Beta	(16)
Risks for main AEs in trastuzumab arm (grade ≥3)					
Neutrophil count decreased	0.11	0.09	0.12	Beta	(16)
Anemia	0.06	0.06	0.07	Beta	(16)
Neutropenia	0.12	0.11	0.14	Beta	(16)
Health state utility in the US					
Progression-free	0.72	0.64	0.79	Beta	(23)
Progressive disease	0.47	0.42	0.52	Beta	(23)
Health state utility in China					
Progression-free	0.85	0.77	0.94	Beta	(24, 25)
Progressive disease	0.52	0.47	0.57	Beta	(24, 25)
Disutility					
Neutrophil count decreased	0.13	0.12	0.14	Beta	(26, 27)
Anemia	0.07	0.07	0.08	Beta	(26, 27)
Neutropenia	0.13	0.12	0.14	Beta	(26, 27)
Discount rate	0.03	0	0.08	Fixed in PSA	-

ASP, Medicare Part B Quarterly Average Sales Price; PSA, probabilistic sensitivity analysis.

Patients in the SOPHIA trial received two treatment regimens, margetuximab plus chemotherapy or trastuzumab plus chemotherapy. Margetuximab was given intravenously at 15 mg/kg each cycle and trastuzumab was given intravenously at 6 mg/kg on day 1 of each cycle after a loading dose of 8 mg/kg. In the base-case analyses, the study modeled patients to remain on treatment unless they were disease-free and did not have a major toxicity event. That implied a median of six cycles for margetuximab vs. five cycles for trastuzumab. Given that, the model adjusted the PFS curve downward by applying the ratio of median time on treatment to median PFS at each cycle. There were four chemotherapy choices including vinorelbine, capecitabine, eribulin, and gemcitabine, with the relative distribution of 35.6, 26.7, 25.4, and 12.3%, respectively (16).

### Costs and utilities

The analyses were conducted from the perspective of the Chinese and US health care system. Direct costs included the drug costs, drug administration, management of adverse events (AEs), best supportive care, and end-of-life care, estimated in 2021 US dollars ($1 = 6.45 Chinese yuan) according to the US and China consumer price index. Drug costs for margetuximab and trastuzumab targeted therapies were derived from projected April 2022 Average Sale Price (ASP) (Genentech data), published literature, and Chinese national drug prices. The US market price of margetuximab was used for the base-case analyses because the margetuximab has not been marketed in China. Drug dosages for margetuximab, trastuzumab, and chemotherapy were based on the SOPHIA trial. The AEs considered in the model were those rated at a severity of grade 3–5 and must have occurred in at least 5% of patients in the clinical trial. The mean cost of AEs for the margetuximab and trastuzumab arms was estimated by multiplying the probability of occurrence of individual AE by the cost of managing each AE. The costs of managing AEs, drug administration and supportive care were estimated based on previous literatures. The study assumed that patients in two groups received best supportive care after progression in the model. Table 1 also includes a summary of cost parameters used in the model.

### Sensitivity analysis

Sensitivity analyses were performed to determine which variables would have a substantial impact on projected costs and outcomes. One-way sensitivity analyses were presented by tornado diagrams. The model also performed probabilistic sensitivity analyses to further test the robustness of the results using Monte Carlo simulation. When the level of confidence was available, variation was based on actual data; when unavailable, the ±20% ranges were assumed for costs, and ±10% for utilities and risks of AEs.

## Results

### Base case

Table 2 shows the detailed information of base-case results. Compared to trastuzumab plus chemotherapy, margetuximab plus chemotherapy were associated with both increased costs and improved outcomes from the US and the Chinese perspectives. From the US perspective, the patients treated with margetuximab plus chemotherapy yielded 0.55 QALYs, with additional 0.09 QALYs than those who received trastuzumab plus chemotherapy. The margetuximab plus chemotherapy costs an additional $23,540, resulting in an ICER of $160,176 compared to trastuzumab and chemotherapy. From the Chinese perspective, margetuximab plus chemotherapy therapy was associated with a mean quality-adjusted survival per patient of 0.65 QALYs, which was 0.11 QALYs longer than trastuzumab plus chemotherapy therapy. The estimated ICER was $630,777 per QALY.

**Table 2:** Discounted incremental cost-effectiveness of margetuximab vs. trastuzumab.

				**Incremental**			**ICER (incremental cost/QALY, $)**
**Analysis**	**Total cost, $**	**LYs**	**QALYs**	**Cost, $**	**LYs**	**QALYs**	
US perspective							
Margetuximab	201,322	0.90	0.55	20,540	0.13	0.09	260,176
Trastuzumab	177,782	0.77	0.46	NA	NA	NA	NA
Chinese perspective							
Margetuximab	106,263	0.89	0.65	68,132	0.12	0.11	630,777
Trastuzumab	38,131	0.77	0.55	NA	NA	NA	NA

LYs, life-years; QALYs, quality-adjusted life-years; ICER, incremental cost-effectiveness ratio; NA, not applicable.

### Sensitivity analyses

**Figure 2:**
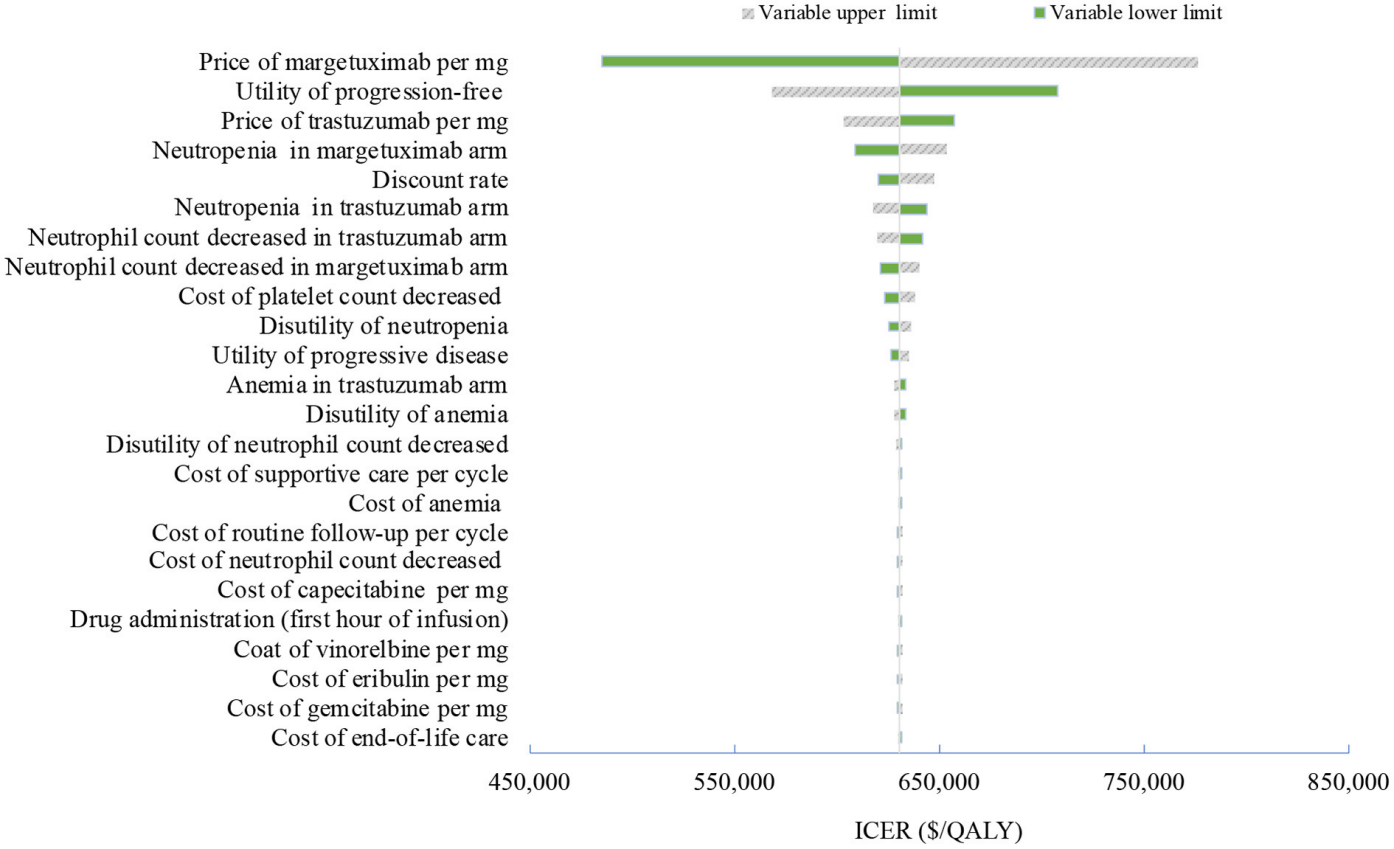
Tornado diagram of one-way sensitivity analyses of margetuximab vs. trastuzumab in the treatment of pretreated ERBB2-positive advanced breast cancer from the Chinese perspective. QALYs, quality-adjusted life-years.

**Figure 3:**
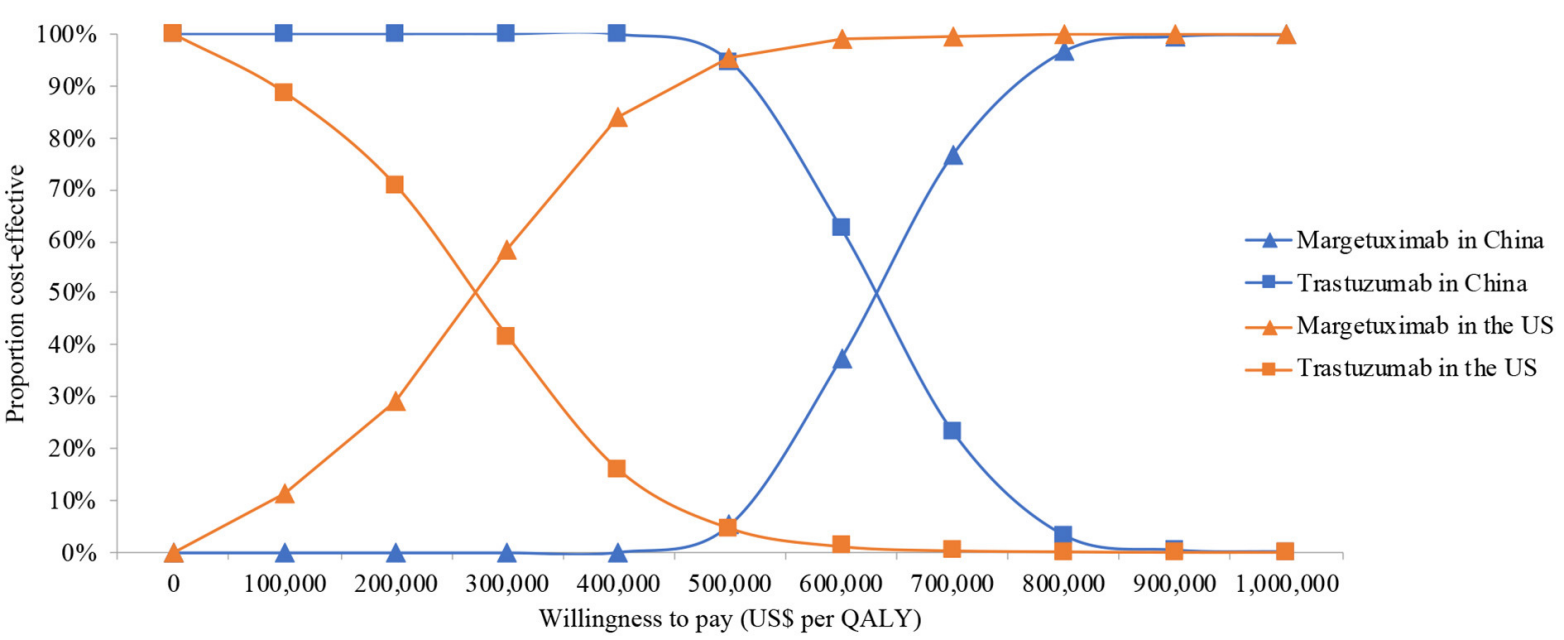
Cost-effectiveness acceptability curve for the base case analysis from the Chinese and the US perspective. QALYs, quality-adjusted life-years.

One-way deterministic sensitivity analyses revealed that the price of margetuximab, the price of trastuzumab altered the cost-effectiveness of the regimens in the US and China (Figures 1, 2), resulting in ICERs varies from $52,913 to $467,436 and from $484,977 to $776,576, respectively. Based on the probabilistic sensitivity analyses (Figure 3), 25% of simulations generated a chance of being cost-effective for margetuximab at a WTP of $150,000, and the percentage would increase to more than 50% at a WTP threshold of $263,000 per QALY in the US. In China, the margetuximab regimen had a 0% chance to be good money for its value at a WTP of $37,653. In addition, the margetuximab regimen was cost-effective when its price was reduced by 11% in the US and 82% in China.

## Discussion

The SOPHIA trial demonstrated a head-to-head advantage of margetuximab compared to trastuzumab, providing a promising option for patients with pretreated ERBB2-positive advanced breast cancer in later line treatment (16). Considering different national conditions and medical environments, this study conducted the model-based cost-effectiveness analyses of margetuximab over trastuzumab, each with chemotherapy, for pretreated ERBB2-positive advanced breast cancer population from the US and the Chinese perspective, leveraging clinical and outcomes data in the SOPHIA trial. Although margetuximab is not available on the Chinese mainland market, the results provided evidence for its pricing in China in the future. The study projected an ICER of $258,147 per QALY gained for the US patients and of $637,656 per QALY gained for Chinese patients. The results support that although margetuximab was associated with improved clinical benefit, it was not cost-effective at the common WTP thresholds of $150,000 and $37,653 in the US and China, respectively. However, it is very close to the cost-effective threshold in the US.

Although no standardized treatment strategies have been established for patients after first-line treatment with ERBB2-positive advanced breast cancer, many candidate third-line and beyond regimens were used historically, including lapatinib with capecitabine, trastuzumab with capecitabine, or other chemotherapeutics with continued trastuzumab (13, 28, 29). Current evidence indicated that the cost-effectiveness was associated with the perspective of studies, the total regimen, and the comparison strategy (30). For example, lapatinib with capecitabine was cost-effective compared to trastuzumab with capecitabine and capecitabine alone from the perspective of the United Kingdom National Health Service (31), while not superior to capecitabine from the US societal perspective (32).

More trials are assessing novel monoclonal antibodies (MoAbs), small molecule tyrosine kinase inhibitors (TKIs), and antibody drug conjugates (ADCs) as the third-line and beyond therapy for ERBB2-positive advanced breast cancer. Margetuximab, the next generation ERBB2-specific MoAbs, resulted in a 1.3-month improvement in median PFS when it replaced trastuzumab in a chemotherapy combined therapy in the third and later lines. Although the base-case analyses failed to prove its cost-effectiveness, sensitivity analyses showed that the result may be reversible when adjusting the price of margetuximab and trastuzumab. A slight decrease in the price of margetuximab would greatly improve its consequence on the value for money in the US. With a 26% price reduction, margetuximab would be dominant over trastuzumab, with a cost-saving and additional QALYs gained in the US, while the price reduction of making margetuximab cost-effective is up to 82% in China. In contrast, the price reduction of trastuzumab reinforces the favorable, cost-effective result itself. The price of trastuzumab in China is 4.5 times cheaper than the US price due to the recent drug negotiation. The nearly identical QALYs gained in the two regimens explained why the cost-effectiveness result is largely dependent on changes in the relative price of margetuximab and trastuzumab. However, since the unsettled optimal treatment paradigm in later lines, margetuximab provides a promising opportunity for patients with pretreated ERBB2-positive advanced breast cancer, especially for those considering the best supportive care.

Recent evidence from the NALA and TULIP trials presents another promising alternative to margetuximab, including the pan-ERBB2 TKI neratinib and ADC (vic-) trastuzumab duocarmazine (SYD985) (33, 34). Substitution of neratinib for lapatinib prolonged median PFS by 2 months (33). Compared with the physician's choice of therapy, a 2.3-month improvement in median PFS was observed for SYD985 (34). The Food and Drug Administration (FDA) approved the neratinib but not yet SYD985 in later-line setting for patients with ERBB2-positive advanced breast cancer in the US. Head-to-head comparison data between margetuximab and other alternatives, along with the cost-efficacy ratios was not available in current evidence, resulting in difficulties in determining the optimal treatment regimen. Despite that, it is reasonable to consider margetuximab as an active regimen for those who are vulnerable to toxic effects of these novel therapies.

This study is subject to limitations. A limitation inherent is reliance on data extrapolation from the clinical trial to a lifetime horizon for economic evaluation. The SOPHIA trial reported the interim analysis results of PFS and OS survival curves. The lack of final survial results reinforces the uncertainty about clinical benefits of the two regimens. If OS in SOPHIA trial is significantly greater than that projected in the present model, the cost-effectiveness of margetuximab will be likely to be improved; however, if margetuximab fails to improve OS, trastuzumab will remain cost-effective. Besides, the quality of life data were available from published literatures rather than from the SOPHIA trial, which failed to reflect the real situation despite conducting sensitivity analyses. Thus, utilities were tested with a range of ±10%, and the result showed that the results were robust.

## Conclusion

The study found that, in patients with pretreated ERBB2-positive advanced breast cancer, despite acceptable safety and significant clinical benefits, margetuximab plus chemotherapy exhibited unfavorable cost-effective result over trastuzumab plus chemotherapy. The cost-effectiveness of margetuximab is very sensitive to the relative price of margetuximab to trastuzumab. The price reduction of margetuximab improves the consequence on its value for money and even makes the regimen cost-effective.

## Author's note

Compared to trastuzumab plus chemotherapy, margetuximab plus chemotherapy has exhibited proven clinical benefits in patients with pretreated ERBB2-positive advanced breast cancer. However, the cost-effectiveness of this new regimen remains to be investigated.

## Data availability statement

The original contributions presented in the study are included in the article/Supplementary material, further inquiries can be directed to the corresponding author/s.

## Author contributions

The conception and design of the study were primarily conducted by YZ and BC. The drafting of the paper was mainly the responsibility of ZT. All authors have reviewed the analysis and interpretation of the data and contributed to the drafting of the manuscript, revising the manuscript for important intellectual content, approved the final version to be published, and agree to be accountable for all aspects of the work. All authors contributed to the article and approved the submitted version.

## Funding

This study was supported by Nantong Science and Technology Plan (MS22020019 and MS22021025), Key Laboratory of New Drug Research and Clinical Pharmacy Research Foundation at Xuzhou Medical University (KFKT-2106), and Nantong Pharmaceutical Society project (ntyx2101).

## Conflict of interest

The authors declare that the research was conducted in the absence of any commercial or financial relationships that could be construed as a potential conflict of interest.

## Publisher's note

All claims expressed in this article are solely those of the authors and do not necessarily represent those of their affiliated organizations, or those of the publisher, the editors and the reviewers. Any product that may be evaluated in this article, or claim that may be made by its manufacturer, is not guaranteed or endorsed by the publisher.
