# Supplementary material for: Economic evaluation of margetuximab vs. trastuzumab for pretreated ERBB2-positive advanced breast cancer in the US and China
Source: Front Public Health. 2022 Sep 9;10:942767. doi: 10.3389/fpubh.2022.942767 (PMC9500445; doi:10.3389/fpubh.2022.942767)
Supplement: Supplementary file 1 [file Data_Sheet_1.DOCX]

# Supplementary materials

| Table S1 Fit Quality (AIC and BIC) for alternat parametric distributions for modelling PFS | | | | |
| --- | --- | --- | --- | --- |
| **Distribution** | **Margetuximab** | | **Trastuzumab** | |
|  | **AIC** | **BIC** | **AIC** | **BIC** |
| Exponential | 1595.92 | 1599.59 | 2394.64 | 2398.79 |
| Weibull | 1524.45 | 1531.80 | 2241.88 | 2250.17 |
| Logistic | 1599.73 | 1607.08 | 2307.42 | 2315.71 |
| Log-normal | **1502.70** | **1510.05** | **2260.28** | **2268.57** |
| Log-logistic | 1512.62 | 1519.97 | 2263.27 | 2271.56 |

Abbreviations: AIC, The Akaike information criterion; BIC, the Bayesian information criterion; PFS, progression-free survival.

| Table S2 Fit Quality (AIC and BIC) for alternat parametric distributions for modelling OS | | | | |
| --- | --- | --- | --- | --- |
| **Distribution** | **Margetuximab** | | **Trastuzumab** | |
|  | **AIC** | **BIC** | **AIC** | **BIC** |
| Exponential | 1364.19 | 1367.79 | 1415.29 | 1418.87 |
| Weibull | 1324.89 | 1332.09 | 1381.62 | 1388.76 |
| Logistic | 1370.29 | 1377.49 | 1424.43 | 1431.57 |
| Log-normal | 1323.44 | 1330.64 | 1392.56 | 1399.71 |
| Log-logistic | **1320.96** | **1328.16** | **1384.32** | **1391.47** |

Abbreviations: AIC, The Akaike information criterion; BIC, the Bayesian information criterion; OS, overall survival.

Table S3 Survival Parameters

| **Parameter** | **Distribution** | **Scale parameter** | **Shape parameter** |
| --- | --- | --- | --- |
| **Progression-free survival** | | | |
| Margetuximab | Log-normal | 1.93 | 0.70 |
| Trastuzumab | Log-logistic | 1.71 | 0.70 |
| **Overall survival** | | | |
| Margetuximab | Log-normal | 21.78 | 2.10 |
| Trastuzumab | Log-logistic | 19.74 | 1.96 |

Table S4 Results of Margetuximab cost discount in China

| **Scenarios** | **Margetuximab cost discount** | | **ICER (USD/QALY)** |
| --- | --- | --- | --- |
| 1 | | 20% off | 484,977 |
| 2 | | 50% off | 266,278 |
| 3 | | 80% off | 47,538 |
| 4 | | 90% off | Dominant |
